# The efficacy of complex decongestive therapy in the treatment of lymphedema associated with endometrial and cervical cancer: evaluation of sensation and balance

**DOI:** 10.1007/s00520-026-10330-9

**Published:** 2026-01-22

**Authors:** Emine Cihan, Cansu Sahbaz Pirincci

**Affiliations:** 1 Department of Therapy and Rehabilitation, Selcuk University, Vocational School of Health Sciences, Physiotherapy Program, Konya, Turkey; 2 Gulhane Faculty of Physiotherapy and Rehabilitation, University of Health Sciences, Ankara, Turkey

**Keywords:** Lower extremity lymphedema, Kinesthesia, Two-point discrimination, Balance, Light touch sensation

## Abstract

**Purpose:**

This study aimed to assess the impact of complex decongestive therapy (CDT) on proprioception, balance, light touch sensation, and two-point discrimination (2PD) in patients with lower extremity lymphedema (LLL) post-endometrial and cervical cancers.

**Methods:**

The study included 72 patients diagnosed with LLL, who were randomly assigned using a block randomization method into two groups: a study group (*n* = 36) receiving CDT and a control group (*n* = 36) receiving no intervention. Patients were assessed before and after treatment using a digital goniometer for proprioception at 15°, 45°, and 60° knee flexion angles, a single-leg balance test with eyes open and closed, and a 30-s chair-stand test for balance assessment. Sensation was evaluated using Semmes Weinstein Monofilaments for light touch sensation and an aesthesiometer for 2PD.

**Results:**

Significant improvements were observed in knee flexion at 15°, 45°, and 60° in the study group compared to the control group (*p* < 0.001, *p* < 0.001, and *p* < 0.001, respectively). Although there was no difference between groups in single-leg balance with eyes open (*p* = 0.074) and closed (*p* = 0.919), improvements were noted within the study group before and after treatment (*p* < 0.001). There was no significant difference between groups in light touch sensation, while the 2PD parameter improved in the study group (*p* = 0.012).

**Conclusions:**

CDT may not fully address sensory deficits in patients with LLL. Balance issues appear to worsen with lymphedema progression regardless of treatment. However, CDT shows promise in improving kinesthesia.

**Clinical Trial Registration:**

This is listed with study ID: NCT06204510.

## Introduction

Lymphedema (LE) is a pathological condition characterized by the accumulation of water, salt, electrolytes, high molecular weight proteins, and other compounds in the interstitial space due to inadequate lymphatic drainage. This condition may be attributed to congenital abnormalities or acquired factors [1]. Affected patients often exhibit increased transepidermal water loss, skin induration, and elevated water content. Hyperkeratotic tissue is a common finding in these individuals. The compromised skin in LE is susceptible to damage, resulting in diminished skin barrier function. This impaired barrier can predispose patients to heightened skin irritation, sensitization, and an increased risk of infection in the affected limb [2].

Affected skin can cause changes in various functions, one of which is proprioception. Proprioception refers to the flow of signals from muscles, tendons, and joints, encompassing both the sense of movement and the ability to position joints (kinesthesia). Loss of proprioceptive or tactile sensory receptors in the extremity leads to a reduction in kinesthetic sensation, thereby impairing the functional capacity of the affected limbs [3]. Balance is a crucial parameter influencing the functionality of the lower extremities. Factors such as cancer diagnoses, chemotherapy, and radiotherapy contribute to reduced mobility and increased clumsiness in the lower extremities. When lymphedema is also present, the negative changes in limb composition further exacerbate these mobility issues [4].

Complex decongestive therapy (CDT) is one of the most important treatment modalities for patients with this clinical condition. However, the treatment of patients still presents many challenges. Research to determine the best treatment to reduce lymphedema, especially in the lower extremities, is lacking in the literatüre [1]. Recent publications on the subject have been criticized for lack of methodological rigor, standardized protocols and lack of controlled trials to compare available treatments, and the predominance of studies focusing on the treatment of upper extremity lymphedema [1, 5].

To the best of our knowledge, studies examining treatment-induced alterations in sensation and balance in lower extremity lymphedema (LLL) resulting from intra-pelvic cancers are scarce in the literature. This study aims to investigate the impact of complex decongestive therapy on proprioception, balance, sensitive tactile sensation, and two-point discrimination (2PD) in patients with LLL following treatment for endometrial and cervical cancers.

## Materials and methods

### Study protocol

The study was designed as a prospective randomized controlled trial to investigate the efficacy of CDT treatment in LLL. It was conducted at a private physiotherapy clinic. Prior to commencement, approval was obtained from the Non-Interventional Ethics Committee of KTO Karatay University (decision no: 2023/021). The study adhered to the principles outlined in the Declaration of Helsinki [6]. After obtaining informed consent from all patients, they were included in the study.

### Patients

Female patients with moderate unilateral lower extremity lymphedema affecting the entire limb—from the foot to the thigh— as a result of endometrial or cervical cancer were enrolled in this study. Patients with edema limited only to the distal part of the limb were not included. The severity of lymphedema was assessed using the criteria defined by the International Society of Lymphology. Specifically, lymphedema was classified as moderate when there was a volume difference of 20–40% in circumference between the affected extremity and the unaffected extremity [7].

**Fig. 1:**
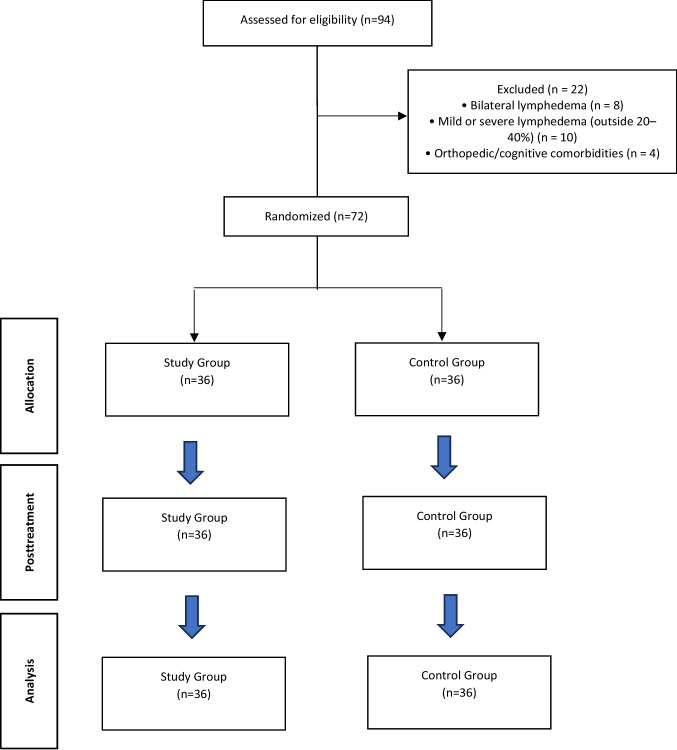
CONSORT flow diagram of the study

A total of 94 patients were screened for eligibility. Of these, 22 were excluded: 8 due to bilateral lymphedema, 10 due to having mild or severe lymphedema outside the 20–40% volume difference range, and 4 due to other exclusion criteria such as orthopedic conditions or cognitive impairment. Ultimately, 72 eligible patients with moderate, unilateral lower extremity lymphedema were enrolled in the study. A total of 72 patients initially participated in the study. These patients were randomly allocated into two groups: a study group and a control group. The final analysis included all 72 patients. The flow diagram of the study is depicted in Figure 1.

In this study, the patients were divided into two groups, and both groups underwent several tests for evaluation before and after treatment. These included the joint position sense test for proprioception assessment, the single-leg balance test and the 30-s chair-stand test for balance evaluation, and the Semmes Weinstein Monofilament assessment and two-point discrimination (2PD) evaluation for sensory evaluation. Demographic data of the patients were collected prior to the commencement of the study.

The inclusion criteria for this study are as follows: participants must have unilateral lower extremity lymphedema resulting from endometrial or cervical cancer, they must have moderate lymphedema as defined by a 20–40% volume difference in circumference between the affected and unaffected extremities, they must be between 18 to 65 years of age, and they must volunteer to participate in the study.

The exclusion criteria for this study encompassed individuals with primary lymphedema, bilateral lymphedema, active infections, mental cognitive impairments, metastases, a history of lower extremity orthopedic surgery, advanced osteoarthritis, joint deformity, or other musculoskeletal conditions that may affect proprioception or joint mobility, and conditions where manual lymphatic drainage is contraindicated (e.g., severe heart failure, thrombosis, or uncontrolled hypertension).

Patients included in the study underwent evaluation upon agreeing to participate in the treatment. The treatment program, specific to study group, spanned a duration of 3 weeks. Final evaluations were conducted at the conclusion of this 3-week period.

The control group consisted of patients who met all inclusion and exclusion criteria but did not receive any therapeutic intervention during the study period. These patients either preferred to postpone treatment for personal, logistical, or financial reasons, were awaiting their scheduled therapy as part of the clinic’s treatment queue, or declined active intervention but agreed to participate in both pre- and post-intervention assessments.

Study termination criteria were established to safeguard the well-being of participants. Treatment or exercise programs would be discontinued if they caused unexpected sensitization or exacerbated symptoms beyond anticipated levels. These criteria were put in place to promptly address any discomfort or complications that might arise during the study, ensuring the highest standards of patient safety and care.

### Randomization and blinding

Participants were divided into two groups: a study group and a control group. The study group received an initial evaluation followed by enrollment in a CDT program. All treatments were administered by a certified lymphedema therapist (EC), while evaluations were conducted by a physiotherapist (CSP), who remained blinded to group allocation and treatment details to ensure impartial assessment. CSP traveled to the site for the scheduled assessment sessions, ensuring consistency in data collection and adherence to blinding.

### Sample size

Power analysis was performed using G*Power (v3.1.9.2, Heinrich-Heine-University, Dusseldorf, Germany) based on mean scores of "Proprioception sense" post-treatment from a study by Cardone (2018) that evaluated proprioception in lymphedema patients. An effect size of 0.897, 95% power, and a 95% confidence interval determined a minimum sample size of 68 participants (34 per group). To accommodate potential data loss during the study, a total of 72 participants were planned for inclusion, representing an approximate 5% increase over the calculated minimum [3].

### Interventions

#### Complex decongestive therapy program

Patients underwent a three-week Complex Decongestive Therapy (CDT) program, involving approximately 45-min sessions five days a week [8, 9]. Each patient received treatment at a scheduled time. The therapy included manual lymph drainage, skin care, compression bandaging, and exercises.

##### Manual Lymphatic Drainage (MLD)

The treatment protocol began with abdominal lymph drainage, followed by central lymph stimulation. Ipsilateral axillary lymph nodes were stimulated to create the axillo-inguinal collateral pathway by extending the patients' knees. Subsequently, the proximal part of the affected extremity was drained first, followed by the distal part. Treatment proceeded from the dorsal to ventral parts of the extremity. The sequence for the proximal part included draining the lateral thigh, ventral thigh, and finally the medial thigh. Moving distally, attention was given to the knee joint, where a lymph pump was created through flexion–extension movements. The lymphedema in the knee was then addressed. Continuing distally, the gastrocnemius muscle was treated using specific techniques, followed by creating a distal pump with ankle dorsiflexion and plantar flexion involving the malleoli. Finally, drainage progressed proximally from the dorsum of the foot to the toes.

##### Skin Care

After manual lymph drainage, a moisturizing cream with a neutral pH, high water content, and low fat content was applied to all lower extremities to alleviate skin tension.

##### Compression Bandaging

After manual lymphatic drainage and skin care, multilayer short-stretch compression bandages were applied to the affected limb to support lymphatic return and reduce edema. Bandaging was performed using a layered technique starting from the toes and progressing proximally, ensuring graded pressure with reduced compression proximally. The bandages were worn for 23 h per day, with 1 h allocated for hygiene and skin inspection. All patients were instructed on proper bandage care and were monitored regularly for signs of discomfort or skin complications.

##### Exercise

After the application of compression bandages, each patient received instructions on daily exercises. Initially, patients performed breathing exercises [9, 10]. These exercises involved abdominal breathing in a supine position with knees flexed, performed 2–3 times daily for 5 min each session.

Following the breathing exercises, patients engaged in exercises to activate the muscle pump. This included ankle dorsiflexion-plantar flexion exercises and cycling movements in the air while in a supine position.

Additionally, weight transfer exercises were conducted using an exercise ball while seated. These exercises were performed 2–3 times daily with 15–30 repetitions per session. Patients were encouraged to briskly walk for 30 min daily as part of their routine. The exercise regimen was repeated daily.

### Outcome measures

#### Assessment of proprioception

##### Joint Position Sense Assessment

Proprioception will be evaluated using the active joint position sense method. This assessment will utilize a digital goniometer [11] to measure knee joint angles separately for each knee.

Target angles of 15°, 45°, and 60° of knee flexion were selected based on commonly referenced values in the literature for evaluating joint position sense across a functional range of motion [11, 12]. These angles represent early, mid-range, and advanced flexion positions and are considered appropriate for proprioceptive assessments of the knee joint in both clinical and research settings.

Participants were assessed in the prone position to minimize the gravitational effect and the influence of limb weight on joint movement. This positioning helps to stabilize the hip and thigh, allowing isolated motion of the knee joint and reducing the contribution of external visual and tactile cues, thereby increasing the accuracy and reliability of proprioceptive measurement.

During the assessment: Participants will first learn the target angle with their eyes open. Subsequently, participants will close their eyes and attempt to replicate the target angle. This process will be repeated three times to familiarize participants with the target angle in the absence of visual input. Upon reaching the target angle in each repetition, participants will hold the position for 10 s to sense the joint position before returning to the starting position. Participants will verbally indicate when they have reached the target angle by saying "here." This will be repeated three times per angle setting, with 5 s of rest between each repetition. The angle displayed on the digital goniometer will be recorded as the actual value, and any deviation from the target angle will be noted as absolute error. Both positive and negative deviations will be considered as positive values. The average absolute error across the three repetitions will be calculated for each angle setting. A 5-min rest period will be provided between assessments of each knee joint [13].

This methodical approach ensures accurate assessment of proprioceptive ability in knee joint position sense under different conditions of visual input.

#### Balance assessment

##### Single-leg balance test

The Single-leg balance test is a straightforward assessment used to evaluate static balance, requiring minimal equipment [14, 15]. Participants will cross their arms in front of their torso and lift one leg so it does not touch the supporting leg. The test will be conducted under two conditions: eyes closed ve eyes open The objective is for participants to maintain balance for up to 30 s without the lifted leg touching the supporting leg, the foot touching the floor, or exhibiting signs of imbalance such as bouncing or touching objects for support. Each condition will be repeated three times for each leg (right and left), and the best result from each set of trials will be recorded.

##### 30-s chair-stand test

The 30-s chair-stand test evaluates lower extremity muscular strength, endurance, and dynamic balance with established validity and reliability [16]. Participants sit in a 43.18 cm (17 inches) high chair with their back straight, feet flat on the floor, and arms crossed. Upon the command "go," they rise to a full standing position and return to sitting as many times as possible within 30 s. Each complete cycle of standing and sitting is counted as one repetition, and the total number of repetitions completed within the timeframe determines the participant's score.

#### Sensory assessment

##### Light touch assessment

The Semmes Weinstein Monofilaments (SWM) is a manual tool utilized for assessing sensory impairments in the skin and gauging the extent of sensory issues stemming from brain injuries [17]. The assessment is conducted with the patient in the supine position. Prior to commencing the test, patients receive an explanation regarding its purpose. The designated test areas include the 1 st and 5th metatarsal heads and the midpoint of the heel. Patients are instructed to avert their gaze from the application site. A monofilament is applied perpendicular to these points and pressed against the skin for 1.5 s, prompting patients to indicate when they perceive the touch by stating "I felt it." Each filament is applied three times within the range of 1.65 to 4.08, and once within the range of 4.17 to 6.65; the monofilament eliciting the correct response is recorded. Responses falling between 1.65–2.83 (green) are classified as indicative of normal touch. Responses within 3.22–3.61 (blue) suggest a mild reduction in tactile sensation. Responses between 3.84–4.31 (purple) indicate decreased protective sensation, while those within 4.56–6.65 (red) denote loss of protective sensation. A lack of response to the filament at 6.65 signifies loss of deep pressure perception [18].

##### Two point discrimination

Participants will undergo assessment while seated and blindfolded, using an aesthesiometer (Instrument Company, Lafayette, IN, USA) [19]. The evaluation will focus on the trans-metatarsal area, midfoot, and mid-heel regions. Initially, the two points will be set at a distance where they are easily distinguishable. Subsequently, the distance between the two points will be gradually reduced in 1 mm increments until the participant perceives them as a single point. At this minimal interval, the two points will be stimulated, and then the distance between them will be incrementally increased by 1 mm intervals until they are once again perceived as two distinct points. Each stimulation of the two points will last approximately 1–2 s, with a waiting period of approximately 3–5 s between stimulations. The shortest distance at which the participant perceives the two points as a single point provides the static two-point discrimination value [20].

### Statistical analysis

Statistical analysis of the study was performed with SPSS program (IBM SPSS Statistics for Windows, Version 22.0. IBM Corp., Armonk, New York, USA). The normality distribution of continuous variables was examined using histogram graphs, skewness and kurtosis coefficients, the Shapiro–Wilk test, coefficient of variance analysis, and detrended normal Q-Q graphs. For the comparison of categorical variables, the chi-square test was used. For the inter-group comparison of continuous variables Mann Whitney U test was used in the case of a non-normal distribution. For the intra-group comparison of continuous variables the Wilcoxon singed-rank test was used in the case of a non-normal distribution. Using statistical software, the effect size was calculated according to the t value and for parametric tests and according to the z value for non-parametric tests. The effect size values of (d) = 0.2, 0.5, and 0.8 and (r) = 0.1, 0.24, and 0.37 were interpreted as small, medium, and large effects, respectively. An overall p-value below 0.05 was considered statistically significant.

## Results

The study was completed with the participation of a total of 72 patients equally distributed in each group. There was no situation that would cause the application of study termination criteria. Patients in the groups were similar in terms of age (*p* = 0.106) and BMI (*p* = 0.698). Patients were homogeneously distributed among the groups in terms of clinical findings. The most common cancer type in both groups (study group = 55.6%; control group = 52.8%) was endometrial cancer. Demographic and clinical data of the patients are given in Table 1.

**Table 1:** Demographic and clinical data of participants

		Study Group (*n*= 36)	Control Group (*n*= 36)		
		Median (IQR)	Median (IQR)	z	p
Age (year)		50 (17)	59 (2)	−1.617	0.106
Height (cm)		159 (6)	160 (15)	−0.364	0.716
Weight (kg)		87 (17)	80 (20)	−1.917	0.055
BMI (kg/m^2^)		34.41 (11.69)	30.89 (1.98)	−0.387	0.698
Duration of Surgery (months)		48 (24)	50 (60)	−1.794	0.073
Duration of Lymphedema (months)		26 (36)	20 (12)	−0.253	0.801
Number of Chemotherapy Sessions		8 (4)	8 (4)	−1.582	0.114
Number of Radiotherapy Sessions		22.5 (10)	28 (10)	−1.403	0.161
		**n (%)**	**n(%)**	**x**^**2**^	**p**
Type of Cancer	Endometrium cancer	20 (55.6)	19 (52.8)	0.056	0.813
	Cervical cancer	16 (44.4)	17 (47.2)		

IQR: Inter Quartile Range, z: Mann Whitney u Test, x^2^: Chi Square Test, *p* < 0.05

In the initial assessment, there were no significant differences in proprioception evaluations between the groups. However, following treatment, there were substantial improvements observed in knee flexion at 15°, 45°, and 60° angles in the study group compared to the control group (*p* < 0.001 for all angles). Specifically, within the study group, there was significant improvement noted in proprioception both before and after treatment (*p* < 0.001) (Table 2).

**Table 2:** Intergroup and intragroup comparison of proprioception assessment

	Study Group	Control Group		
	**Median (IQR)**	**Median (IQR)**	**z**	**p**
Pre-Treatment Proprioception 15°	24 (7.25)	23.83 (4)	−1.437	0.151
Post-Treatment Proprioception 15°	19 (4.17)	23 (3)	−7.369	** < 0.001**
p	** < 0.001**	0.145		
z	−5.263	−1.457		
Effect size	0.877	0.243		
Dif	6 (3.75)	0 (0.33)	−6.829	< 0.001
Pre-Treatment Proprioception 45°	52.66 (3.75)	54 (0.33)	−0.217	0.828
Post-Treatment Proprioception 45°	42 (8)	54 (12)	−5.144	** < 0.001**
p	** < 0.001**	0.096		
z	−5.273	−1.666		
Effect size	0.879	0.278		
Dif	10 (3.33)	−2.33 (3.67)	−6.732	< 0.001
Pre-Treatment Proprioception 60°	68 (3.33)	74 (3.67)	−2.375	0.018
Post-Treatment Proprioception 60°	62 (15)	75 (11)	−3.833	** < 0.001**
p	** < 0.001**	0.154		
z	−4.350	−1.427		
Effect size	0.725	0.238		
Dif	6 (20)	−1 (1)	−5.943	< 0.001

Dif: Differences, IQR: Inter Quartile Range, z: Mann Whitney u Test, *p* < 0.05

Comparing the balance evaluations of the patients standing on single leg, no significant differences were found between the groups in the parameters of single leg eyes open and single leg eyes closed (*p* = 0.074 and *p* = 0.919, respectively). However, there was a notable improvement in the single-leg balance test with eyes open parameter observed in the study group after treatment (*p* = 0.05, z = −2.883). In the 30-s chair-stand test, there was a significant increase in the number of sit-to-stand repetitions both within and between groups after treatment (*p* < 0.001 for both comparisons) (Table 3).

**Table 3:** Intergroup and intragroup comparison of balance assessment

	Study Group	Control Group		
	**Median (IQR)**	**Median (IQR)**	**z**	**p**
Pre-Treatment Single-leg balance (eyes open)	5.86 (10)	7.35 (5)	−1.634	0.102
Post-Treatment Single-leg balance (eyes open)	19.15 (34.13)	10 (33.55)	−1.788	0.074
p	**0.005**	0.074		
z	−2.833	−1.785		
Effect size	0.472	0.298		
Dif	−0.46 (2.95)	0.00 (3.50)	−1.063	0.288
Pre-Treatment Single-leg balance (eyes closed)	5.5 (2.95)	7.5 (3.5)	−0.045	0.964
Post-Treatment Single-leg balance (eyes closed)	5.5 (21.5)	7 (17)	−0.102	0.919
p	0.589	0.947		
z	−0.540	−0.066		
Effect size	0.090	0.011		
Dif	0.00 (1.75)	0.00 (1)	−0.119	0.905
Pre-Treatment 30-s chair-stand test	14 (1.75)	12 (1)	−0.926	0.354
Post-Treatment 30-s chair-stand test	16 (5)	13 (5)	−3.561	** < 0.001**
p	** < 0.001**	0.089		
z	−3.973	−1.701		
Effect size	0.662	0.284		
Dif	−4 (7.25)	0.050 (2)	−2.415	0.016

Dif: Differences, IQR: Inter Quartile Range, z: Mann Whitney u Test, *p* < 0.05

When comparing the study and control groups in terms of light touch sensation, no statistically significant differences were observed at the first Metatarsophalangeal Joint, fifth Metatarsophalangeal Joint, and heel points (*p* = 0.862, *p* = 0.221, and *p* = 0.748, respectively). Similarly, intra-group evaluations did not reveal any significant differences. Regarding the two-point discrimination parameter, there was a significant increase in values within the study group (*p* = 0.012). In the intergroup comparison, the study group also demonstrated a significant increase compared to the control group (*p* = 0.006) (Table 4).

**Table 4:** Intergroup and intragroup comparison of sensory assessment

	Study Group	Control Group		
	Median (IQR)	Median (IQR)	z	p
Pre-Treatment 1 st MTP Joint	3.22 (20)	2.83 (1)	−0.961	0.336
Post-Treatment 1 st MTP Joint	3.22 (0.78)	3.22 (1.17)	−0.173	0.862
p	0.636	0.767		
z	−0.473	−0.296		
Effect size	0.078	0.049		
Dif	0.00 (0.78)	−0.19 (1.40)	−0.756	0.450
Pre-Treatment 5th MTP Joint	3.22 (0.78)	3.22 (1.4)	−1.425	0.154
Post-Treatment 5th MTP Joint	3.22 (0.63)	3.22 (1.07)	−1.223	0.221
p	0.944	0.905		
z	−0.070	−0.120		
Effect size	0.011	0.020		
Dif	0.00 (0.00)	0.00 (0.00)	−0.242	0.809
Pre-Treatment midheel	3.84 (0)	3.84 (0)	−0.429	0.668
Post-Treatment midheel	3.84 (0.23)	3.84 (0.52)	−0.321	0.748
p	0.141	0.496		
z	−1.473	−0.680		
Effect size	0.245	0.113		
Dif	0.00 (0.00)	0.00 (0.00)	−0.98	0.327
Pre-Treatment 2PD	15 (0)	15 (0)	−0.382	0.703
Post-Treatment 2PD	14 (2)	16 (2)	−2.739	**0.006**
p	**0.012**	0.828		
z	−2.513	−0.217		
Effect size	0.418	0.036		
Dif	1.50 (3)	−1 (3.75)	−2.412	0.016

*MTP* Metatarsophalangeal, *2PD* Two Point discrimination, *Dif* Differences, *IQR* Inter Quartile Range, *z* Mann Whitney u Test, *p* < 0.05

## Discussion

This study was conducted to investigate the effects of CDT treatment on proprioception, sensation and balance in patients who developed lymphedema in the lower extremities due to endometrial and cervical cancers. Lymphedema represents a comprehensive challenge for affected individuals. Proprioception assessments at various angles demonstrated significant improvements among patients undergoing treatment. Although patients receiving treatment showed enhanced balance in single-leg positions, these improvements did not reach statistical significance compared to the untreated group. The patients' repetitive sit-to-stand performance in 30 s increased with the treatment. There were no observed improvements in light touch sensation among the patients; however, notable enhancements were noted in two-point discrimination abilities.

Cardone et al. reported in their kinesthesia study that proprioception impairment in women with upper extremity lymphedema was not correlated with limb circumference. Nevertheless, they noted that reduced proprioceptive sensation in the affected extremity significantly impacted activities of daily living [3]. In a study by Zabir et al. (2023), it was demonstrated that mastectomy and adjuvant therapies had an effect on shoulder receptors and resulted in impaired proprioception in cases of upper extremity lymphedema [21]. In the existing literature, we did not identify studies specifically comparing proprioception assessments in patients with LLL. Our study findings indicate that proprioception was impaired during knee flexion angles in LLL patients. However, we observed a recovery in proprioceptive sensation among patients receiving CDT. Specifically, the ability to accurately reposition the limb at these angles improved by approximately 22% at 15°, 19% at 45°, and 9% at 60° among treated patients. The compromised kinesthesia and proprioception in these patients may be attributed to the nature of lymphedema. Accumulation of subcutaneous fat and fibrosis associated with lymphedema likely diminishes the activity of proprioception receptors, thereby reducing kinesthetic sensation. Similar conclusions can be drawn from studies demonstrating reduced knee kinesthesia in obese individuals, where increased subcutaneous adipose tissue impairs proprioception receptors and leads to diminished postural control. Consequently, these factors contribute to posture disorders [22].

It is recognized that proprioception plays a crucial role in both static and dynamic postural stabilization, as well as joint stabilization. Proprioceptive signals from receptors in the lower extremities are essential for regulating the foot swing [23]. In individuals with higher BMI, there is notable variation in the sensitivity of plantar mechanoreceptors. This sensitivity variation impacts several aspects, including balance and muscle strength [24]. Considering the increase in body weight in lymphedema, this could contribute to the affected receptors. In the sensory tests we conducted, the treatment group showed a positive improvement in two-point discrimination, indicating that treatment may have slightly improved the extremity affected by the fibrotic structure of skin receptors and edema. The patients' inability to sense mild sensations is a consequence of the underlying pathology, independent of whether they receive treatment. Ultimately, lymphedema significantly impacts extremity tissues. Additionally, chemicals and radiation from chemotherapy and radiotherapy treatments may have affected the skin and its receptors.

Doruk Analan ve ark. found that balance impairment in the lower extremities was more pronounced in individuals with LLL compared to healthy controls. However, this did not necessarily elevate the risk of falling [25]. Similarly, visual inputs (eyes open vs. closed) and ground support (single foot vs. double foot) significantly exacerbate impaired balance in LLL patients, affecting both static and dynamic balance when compared to healthy individuals. Additionally, Karasimav et al. highlighted that proprioceptive signals play a paramount role in balance, exerting a greater influence than visual and vestibular components [26]. In this study, reduced ground support and diminished visual input were observed to increase the volume of lymphedema in the affected extremity, thereby compromising the patient's static balance. Moreover, the enhancement of proprioception following treatment in the study group contributed to intra-group balance improvements. Despite improvements across various parameters due to treatment, the persistence of pathology may have contributed to decreased balance. Patients' difficulty in adjusting to their altered body composition post-treatment and the potential lack of improvement from bandaging sensation could have hindered balance enhancement. Furthermore, performance in sit-to-stand tests was negatively impacted in the untreated LLL group, affecting dynamic balance and functionality. Enhanced proprioception and foot sensation positively influenced functionality. Additionally, changes in foot size and functionality also influenced balance outcomes [27].

This study has several limitations that should be acknowledged. the study did not inquire about the patients' pre-existing balance issues, which could have influenced the baseline measurements. Future studies could benefit from employing advanced measuring devices to obtain more objective and precise data.

The findings of the study highlight the complex nature of lymphedema and suggest that Complex Decongestive Therapy (CDT) may offer benefits by improving proprioception and two-point discrimination in affected patients. However, further research is necessary to better understand the mechanisms underlying these improvements and to optimize therapeutic strategies for the comprehensive management of lymphedema-related impairments.

To our knowledge, this study represents a rare focus on sensation, proprioception, and balance in patients with LLL. In conclusion, CDT the gold standard for lymphedema treatment, has demonstrated significant effectiveness in improving various outcomes. However, there may be room for protocol refinement, particularly concerning patients' challenges with static balance and reduced mild tactile sensation. Integrating balance exercises and sensory integration therapies into the rehabilitation protocols for LLL patients could potentially enhance treatment outcomes. This approach may address residual impairments and further optimize the management of lymphedema-related symptoms. Future research should explore these interventions to refine therapeutic strategies and improve overall patient care.

## Data Availability

Not applicable.
